# Case Report: Cannabis and kratom-induced self-amputation of ears and penis

**DOI:** 10.3389/fpsyt.2025.1479863

**Published:** 2025-03-18

**Authors:** Marek Broul, Xenia Rudenko, Adam Bajus, Jiří Král, Dan Mwemena Kyenge, Zdenka Staňková, Jakub Albrecht

**Affiliations:** ^1^ Department of Sexology, Krajská Zdravotní, Masaryk Hospital, Ústí nad Labem, Czechia; ^2^ Department of Urology, Krajská Zdravotní., Hospital Litoměřice, Litoměřice, Czechia; ^3^ Faculty of Health Studies, Jan Evangelista Purkyně University, Ústí nad Labem, Czechia; ^4^ Psychiatric Clinic of The Faculty of Health Studies, Jan Evangelista Purkyně University, Krajská Zdravotní, Masaryk Hospital, Ústí nad Labem, Czechia; ^5^ Department of Reconstructive and Plastic Surgery, Krajská Zdravotní, Masaryk Hospital, Ústí nad Labem, Czechia; ^6^ Department of Urology, Most Hospital, Krajska Zdravotni, Most, Czechia; ^7^ Department of Psychiatry, Most Hospital, Krajska Zdravotni, Most, Czechia

**Keywords:** case report, substance-induced psychosis, self-inflicted injuries, self-amputation, cannabinoids, kratom, psychotic episodes, paranoia

## Abstract

This report describes the case of a 31-year-old male lumberjack with severe self-inflicted injuries, including the amputation of both auricles and the penis, under the influence of cannabinoids, mitragynine, and 7-hydroxymitragynine. Emergency surgery was performed, and psychiatric evaluation revealed substance-induced psychosis. The patient’s motivation for reconstructive penile surgery led to abstinence from the substance use and cooperation with treatment. Five months after hospitalization, successful penile reconstruction was completed. The patient remained abstinent and was engaged in regular psychiatric follow-ups, showing no signs of acute psychopathology. This case underscores the importance of using a multidisciplinary approach to manage severe self-harm behaviors, and highlights the critical role of patient motivation in achieving positive outcomes.

## 1 Introduction

Self-inflicted injuries are rare but severe manifestations of psychiatric and substance use disorders. These behaviors range from superficial cuts to more extreme forms including self-amputation of body parts. This case report describes a rare instance of self-inflicted ear and penile amputation in a 31-year-old man who was under the influence of multiple psychoactive substances, including cannabinoids, mitragynine, and 7-hydroxymitragynine.

Psychotic episodes induced by drug use may lead to severe self-harm including genital mutilation (1). These behaviors are often associated with psychosis, where individuals may experience delusions or hallucinations that drive them to harm themselves (2). The involvement of substances, such as mitragynine and 7-hydroxymitragynine, which are compounds found in kratom, adds a unique aspect to this case, highlighting the potential psychiatric risks associated with their use.

Self-harm involves deliberately causing harm to one’s own body as a way to cope with overwhelming emotions or psychological pain. Common forms of self-harm include cutting, burning, scratching, or hitting oneself, although it can also manifest in more indirect behaviors such as reckless substance use or other high-risk activities. In psychiatry, self-harm often coexists with conditions like depression, borderline personality disorder, or acute stress reactions, and serves as a maladaptive coping mechanism for emotional regulation. Substance abuse can exacerbate the risk of self-harm by impairing judgment, increasing impulsivity, or intensifying emotional distress, thereby lowering the threshold for self-injurious behavior.

This case report was prepared according to the CARE Guidelines (3, 4). The report aims to provide a detailed account of the patient’s presentation, clinical management, psychiatric evaluation, and follow-up, emphasizing the importance of recognizing and addressing the psychiatric dimensions of substance-induced psychosis and self-harm.

## 2 Case description

### 2.1 Patient information

The patient was a 31-year-old Caucasian man who was employed as a lumberjack. He presented with severe self-inflicted injuries, including the amputation of his penis and both auricles, multiple lacerations on his forearms, and frostbite on his feet and toes. The injuries were sustained under the influence of multiple psychoactive substances.

#### 2.1.1 Medical history

The patient had a history of substance abuse, including regular consumption of approximately 1.5 L of beer every other day, daily marijuana use (approximately 1 g/day, which had been reduced to every other day over the preceding 2 years), and occasional use of methamphetamine and psilocybin mushrooms. He had previously experienced psychotic episodes associated with his substance abuse. The patient did not take any prescribed medications and was not being treated for any chronic conditions. He had recently been diagnosed with prediabetes.

The patient’s first contact with alcohol occurred around the age of 15. He reports never consuming alcohol in the morning, but mentions episodes of short-term memory lapses (“blackouts”) following hard liquor. He started using THC (cannabis) at about 17 years of age; currently, he states that over the past two years he has reduced his use to approximately one “joint” every other day, primarily in the evening before bedtime (previously it could be one or two joints daily). He first tried methamphetamine (pervitin) at 19 but only on two or three occasions in total. He denies any intravenous use. He used hallucinogenic mushrooms (psilocybin) for the first time at 18, with an estimated total of about 10 episodes (most frequently in the fall). He also reports occasional use of other hallucinogens, such as LSD at music festivals, but cannot specify frequency or exact timing. He first tried kratom about a year ago and finds it difficult to specify how often he has used it. The patient acknowledges frequent combination of alcohol with other substances; due to recurring memory lapses, he often cannot be certain which substances he consumed or in what quantity.

For most substances (THC, hallucinogens, kratom), the patient describes oral or inhalation routes. He explicitly denies intravenous use (e.g., of methamphetamine). There is no record of formal, extended abstinence periods; the patient only mentions occasional “breaks” in cannabis and alcohol use in the past, without formal treatment or therapy.

Medical records indicate that the patient engaged in self-harm in the context of mixed intoxication (alcohol, cannabis, psilocybin) in 2018. He was hospitalized following an episode of aggressive outburst and self-harm behavior, Documentation from this hospitalization mentions, among other findings, vulnera scissa in the thoracic and abdominal wall regions, a frontal hematoma, and bite wounds to the lips and tongue.

During the aforementioned hospitalization, no florid psychotic symptoms persisted once acute intoxication resolved. Consequently, there was no specific psychiatric treatment for psychosis, and the patient was discharged without antipsychotic medication. No further specialized psychiatric treatment for drug-induced psychotic symptoms is noted in the available documentation.

The patient had his first more serious relationship in high school, followed by several long-term relationships in adulthood (lasting 4, 5, 3, and 0.5 years). The most recent relationship ended about a year ago due to long-distance issues. He reports first sexual intercourse around the age of 17.

#### 2.1.2 Family history

Both the patient’s parents had a history of nicotine dependence and alcohol abuse. No other notable family medical conditions were reported by the patient.

#### 2.1.3 Psychiatric history

During this episode, the patient experienced significant psychomotor agitation and paranoid delusions. His emotional response to his injuries and situation was inappropriate, indicating a lack of interest in the treatment or consequences. Psychosocial examinations revealed low intellectual performance, impaired social judgment, and an inability to control emotions. He denied regular use of other substances but admitted to the recent use of multiple drugs.

The patient had been hospitalized previously because of aggressive behavior and self-harm associated with substance use. These interventions included psychiatric evaluation and treatment, which led to temporary stabilization. However, he had not maintained long-term follow-up or adhered to any prescribed psychiatric or medical treatment, which resulted in recurrent episodes of substance-induced psychosis and self-harm.

### 2.2 Clinical findings

On admission, the patient underwent a comprehensive diagnostic assessment. Physical examination revealed severe self-inflicted injuries, including the amputation of both the auricles and penis, multiple lacerations on the forearms, and frostbite on the feet and toes. He was hemodynamically stable and exhibited no signs of acute distress other than the visible injuries. This self-inflicted injury occurred in January when it was freezing and temperatures reached minus 7 degrees Celsius, leading to frostbite of the lower extremities. We do not know the time interval after the patient was found after the automutilization, but apparently due to the freezing weather there was no massive bleeding and death. He was conscious, but displayed significant psychomotor agitation and paranoia. Despite cooperating during the physical examination, he lacked awareness of the severity of his injuries.

Initial laboratory tests were conducted to evaluate the patient’s overall health status and detect any potential infections. Toxicological screening confirmed the presence of cannabinoids, mitragynine, 7-hydroxymitragynine, and other compounds. Imaging studies included computed tomography (CT) of the head and abdomen, which ruled out intracranial bleeding and intra-abdominal injuries, and indicated no acute abnormalities.

According to the available toxicological findings immunochemical testing of the urine detected the presence of cannabinoids as a group (without specifying the exact derivative). Subsequent mass spectrometry explicitly confirmed morphine, mitragynine, and 7-hydroxymitragynin, but it did not provide detailed quantification or identification of the specific cannabinoid. In standard toxicological practice, this typically indicates the detection of THC metabolites (especially 11-nor-9-carboxy-THC), although in this instance, the mass spectrometry did not specify which particular cannabinoid was found.

### 2.3 Diagnostic assessment and diagnosis

The patient underwent detailed psychiatric evaluation, which revealed significant psychomotor agitation and paranoia. He was cooperative during the examination but lacked awareness of the severity of his injuries. The results of the comprehensive psychological examination indicated low intellectual performance, impaired social judgment, and an inability to control emotions. The patient also displayed negative self-assessment and had difficulty identifying and fulfilling his own needs. He did not provide insights into his psychological or somatic state, or substance use.

As part of a comprehensive clinical evaluation, psychological screening was conducted using both standard observation and interview techniques as well as specific psychodiagnostic methods. The following tools were used: observation and clinical interview, WAIS-III (Wechsler Adult Intelligence Scale, 3rd edition), ROCFT (Rey–Osterrieth Complex Figure Test), TMT (Trail Making Test), VF (Verbal Fluency), ROR (Rorschach Test), Baumtest (Tree Drawing Test) and Human Figure Drawing. All these instruments contributed to assessing the patient’s cognitive performance, executive functions, emotional experience, and personality traits. Based on the findings, the patient demonstrates below-average intellectual performance (not reaching the threshold of mental retardation), impaired social judgment, and reduced verbal and mental flexibility. Although there are no signs of a florid psychotic disorder, discrete perceptual and thought disturbances, difficulties with affect control, and a lack of insight into both his substance use and current psychological state are evident. A sexological evaluation was performed, during which the patient expressed acceptance of his condition and identified himself firmly as a male with no desire to change gender. He reported several long-term relationships with women and denied any homosexual encounters, although he acknowledged the difficulty of remembering all past experiences.

The differential diagnosis was substance-induced psychosis, given the patient’s history of substance abuse and the presence of multiple psychoactive substances. A diagnosis of F19.5 psychotic disorder owing to multiple substance use was established. This was supported by the temporal relationship between substance use and the onset of psychotic symptoms as well as the patient’s history of similar episodes. At the age of 24, the patient had experienced a similar episode in which he became aggressive and self-harmed by cutting his chest after using alcohol, marijuana, and psilocybin mushrooms. The psychotic symptoms had resolved after detoxification, and the patient was discharged after several days.

Primary psychotic disorders, such as schizophrenia, were also considered but deemed less likely due to the temporal relationship between the substance use and the onset of psychotic symptoms. Organic causes were ruled out on the basis of negative CT imaging results and normal laboratory findings. Given the history of substance abuse, the diagnosis of an acute psychotic disorder (F23.x) was also considered, but was less likely. However, the patient’s sensitivity to the development of psychotic processes indicated a potential risk of future schizophrenic spectrum disorders if substance use continued.

The ultimate primary diagnosis was substance-induced psychosis with severe self-harm. It was supported by the patient’s history of similar episodes of substance use. Prognostically, patient recovery depends on their ability to abstain from substance use, adhere to psychiatric follow-up, and undergo successful surgical and rehabilitative interventions for their physical injuries.

### 2.4 Patient case timeline

The patient case timeline is presented in **Table 1**. The patient was hospitalized for a total of 31 days, broken down as follows: 2 days in the urology department and 29 days in the psychiatric ward (including 12 days in a psychiatric detox bed – ICU). The entire hospitalization was involuntary and was reported to the court in accordance with the relevant legal framework.

**Table 1:** The patient case timeline.

Day	Event
0	Self-inflicted injuries (amputation of penis, auricles, forearm lacerations)
1	Admitted to the emergency department in Most
1	Epicystostomy, revision of penile stump and auricle amputation defects
1	Psychiatric consultation - diagnosed with psychosis
1	Involuntarily transferred to Psychiatric Clinic
16	Underwent sexological and psychological evaluations
19	Subsidence of psychosis symptoms, voluntary admission, increased cooperation
21	Consultation with a plastic surgeon
28	Discharged for regular outpatient psychiatric care
71	Outpatient consultation with plastic surgeon
151	Admitted for penile reconstructive surgery
170	Discharged post-surgery

### 2.5 Therapeutic interventions

On admission, emergency surgery was performed to manage the amputation of the penis and both auricles. The procedure included hemostasis and wound debridement. Because the amputated parts were not recovered, replantation was not possible. Broad-spectrum antibiotics were administered to prevent infection, analgesics were administered for pain management, and sedatives were used to control psychomotor agitation and paranoia.

Detailed psychiatric evaluation revealed significant psychomotor agitation and paranoia. Treatment with olanzapine (10 mg/day) was initiated to manage the psychotic symptoms and diazepam (5 mg as needed, up to 30 mg/day) for the anxiety and agitation. Owing to the patient’s psychotic behavior, initial sedation with standard doses was insufficient. The olanzapine dosage was consequently increased to 15 mg/day, and the diazepam dosage was adjusted to ensure adequate sedation and psychotic symptom control.

During the hospital stay, the patient developed frostbite on his feet and toes. Conservative management, including wound care and monitoring for signs of infection or necrosis, was performed. The patient’s psychiatric medications were regularly reviewed and adjusted based on his response to treatment, leading to gradual stabilization of his mental state. The patient was presented to a plastic surgeon and informed of the possibility of surgical penile and auricular reconstruction. However, reconstruction was contingent on patient cooperation and full abstinence from substance use.

A mental capacity assessment was conducted. This evaluation included a comprehensive psychological examination using the WAIS-III scale, which indicated that the patient’s current intellectual performance is in the below-average range (with no signs of mental retardation). The individual indices (Verbal Comprehension, Perceptual Organization, Working Memory) also fall within the below-average range, while only Processing Speed lies on the borderline between average and below average.

Moreover, the patient exhibits significantly impaired social judgment, limited knowledge of common behavioral norms, and reduced judgment in practical social situations. The weighted scores across both verbal and performance subtests predominantly fall into the below-average range. Based on these findings, the patient demonstrates reduced cognitive abilities in several areas, which may affect his capacity to adequately assess his actions and make decisions; however, a formal conclusion regarding legal capacity would require a comprehensive evaluation and potential further legal assessment by the court.

### 2.6 Follow-up and outcomes

Throughout the patient’s hospital stay and subsequent follow-up, both clinician- and patient-assessed outcomes were closely monitored. The patient’s physical condition, including the surgical sites for the amputations and frostbite on the feet and toes, was regularly evaluated for signs of infection and healing progress. Psychiatric evaluations were conducted to manage the psychomotor agitation and paranoia, leading to necessary medication adjustments.

The patient was initially compliant with treatment using olanzapine and diazepam, which helped stabilize his psychiatric symptoms. However, he refused institutional treatment for substance abuse, thus minimizing its significance. Despite the resolution of psychotic symptoms, he did not gain complete insight into his condition. He was discharged on the 29th day of psychiatric hospitalization with an outpatient follow-up plan.

Five months after the initial hospitalization, the patient underwent reconstructive penile surgery. He was highly motivated and involved in the planning of the surgery; he showed a strong desire for reconstruction and cooperated closely with the medical team. During his hospital stay, a psychiatrist assessed his mental state. The patient denied the use of any addictive substances, as proven by blood tests and reported no psychological issues. He stated that he had adhered to his medication regimen after discharge and had only recently discontinued antipsychotics. He also mentioned that he regularly attended psychiatric check-ups to regain his driver’s license. The patient showed no signs of acute psychopathology.

Given the patient’s refusal to undergo substance abuse treatment and psychiatric follow-up, a coordinated approach involving social services and outpatient psychiatric care was recommended to support his post-discharge transition. The patient was informed of the critical importance of adhering to the medical and psychiatric advice to prevent the recurrence of severe psychotic episodes and self-harm.

The available information suggests that abstinence began after the patient’s discharge into outpatient psychiatric care; however, the exact date and the duration of abstinence are not precisely documented. During this period, abstinence was monitored primarily through clinical examination, the patient’s own reports, and bedside saliva screening tests. There is no specific data regarding any blood test that may have been used; therefore, it is not possible to state which particular blood screening test was employed in this case.

The patient was offered psychosocial support by the local community mental health center team, which he declined, as well as recommended outpatient addiction treatment, which he also refused. Currently, he only attends appointments with an outpatient psychiatrist.

## 3 Patient perspective

“Looking back on that night, in January 2024, I felt completely overwhelmed and acted on a sudden urge to hurt myself. I ended up severely injuring myself by cutting off parts of my body. The next thing I remember is being rushed to the hospital, where doctors performed several procedures to treat my injuries. Later, I was told that I had experienced a psychotic episode and was moved to a psychiatric clinic. The first few days were a blur of medical tests and assessments. Gradually, with the help of the medical team, I began to feel more connected to reality. By early February, I started feeling better and agreed to participate in my treatment. This included meetings with a plastic surgeon and receiving regular psychiatric care. Before my reconstructive surgery in June, I struggled with epicystostomies. This made it impossible for me to work in the forest, and I lost my job because of it. The surgery was a major step in my recovery. Now I’m out of hospital and getting regular outpatient care. I have also decided to stop using drugs, as I know they contributed to my breakdown. I’m working on getting my driver’s license back so that I can regain my independence and function better in daily life. I feel more hopeful and committed to my mental health journey.”

## 4 Discussion

This case report illustrates the complex interplay between substance-induced psychosis and severe self-harming behaviors, highlighting the necessity for a multidisciplinary approach to both acute and long-term management. A significant strength of this case was the rapid and coordinated surgical intervention to manage the severe self-inflicted injuries and prevent life-threatening complications. Comprehensive psychiatric and psychological evaluations provided critical insights into the patient’s mental state and guided subsequent treatment. However, a major limitation was the patient’s refusal to engage in substance abuse treatment and non-compliance with psychiatric follow-up, which significantly affected his prognosis.

The use of psychoactive substances has increased significantly worldwide. New substances emerge almost daily, contributing to a broader spectrum of behavioral disorders and acute psychopathology than traditional drugs. With the development of designer and synthetic drugs, an increase in problems associated with their abuse can be anticipated (5).

Substance-induced psychosis leading to self-mutilation has been well documented. Khan et al. (6) presented a case of self-amputation of the penis owing to cannabis-induced psychosis, highlighting the severe self-harming behaviors associated with substance use disorders. Jones (7) reported cases of self-enucleation in patients with drug-induced psychosis and schizophrenia, underscoring the psychiatric complexities involved. Other notable cases include a report on radical facial self-mutilation, by Scheftel et al. (8), which highlighted unprecedented self-harming behaviors. Coons et al. (9) documented the self-amputation of the female breast, demonstrating the severity of self-inflicted injuries in psychotic patients.

The case reported by Koops and Püschel (10) involved a patient with paranoid-hallucinatory schizophrenia who self-amputated both auricles and the glans of the penis and ingested the amputated parts. This is the only other published case in which both the auricles and the penis were amputated. In this case, the patient consumed the amputated parts, and the injury led to severe blood loss and death. The auricles and penis are highly vascularized, making these injuries life-threatening, owing to potentially significant blood loss. Fortunately, our patient survived, probably because of vasoconstriction from the cold, and timely medical intervention.

The primary diagnosis of substance-induced psychosis was supported by the temporal relationship between the substance use and the onset of psychotic symptoms, as well as the patient’s history of similar episodes. Refusal to engage in substance abuse treatment and psychiatric follow-up posed significant challenges, highlighting the need for a more integrated approach involving social services and continuous psychiatric care. Interestingly, the possibility of penile reconstruction and the aim of regaining a driver’s license served as strong motivations for the patient to maintain abstinence during the follow-up period. His intense involvement and cooperation in the planning of the reconstructive surgery contributed significantly to the positive outcome, demonstrating that patient motivation could be a critical factor in treatment success.

Cannabis, particularly in high-THC formulations, can contribute to acute psychosis through dysregulation of dopaminergic signaling in brain regions associated with reward and cognition (e.g., the mesolimbic pathway). Tetrahydrocannabinol (THC) acts as a partial agonist at cannabinoid CB1 receptors, which can secondarily increase dopaminergic release, potentially exacerbating paranoid thinking, delusional ideation, and risk of self-harm in susceptible individuals. This effect is further influenced by genetic predispositions, baseline mental health status, and environmental stressors (11).

Kratom’s primary alkaloids—mitragynine and 7-hydroxy-mitragynine—exhibit partial agonism at µ-opioid receptors and may modulate other neurotransmitter systems (e.g., adrenergic, dopaminergic). In higher doses or in individuals with certain vulnerabilities, the resulting neurochemical imbalance can provoke mood dysregulation, impaired judgment, and—in rare cases—acute psychotic features. Although psychotic reactions to kratom are less well-characterized than those to cannabis, potential mechanisms include opioid receptor-mediated changes in dopamine turnover and heightened stress responses, creating a fertile ground for hallucinations, delusional thinking, and self-harm behaviors (12).

Precise data on acute psychosis or delusional states specifically leading to self-harm after cannabis or kratom use are lacking. For cannabis, some registries estimate around 1–5 cases of acute psychosis per 100,000 population per year, but direct links to self-harm within these episodes remain unclear. For kratom, robust incidence rates do not exist due to scarce epidemiological research and reliance on isolated case reports (13).

Emerging evidence underscores that while acute psychosis and self-harm can occur with either substance, no clear, universally accepted incidence rate exists due to methodological limitations and a lack of large-scale prospective studies. As a result, clinicians and public health professionals typically rely on case reports, smaller observational studies, and anecdotal evidence when assessing risk. (14)

Continuous follow-ups and adherence to psychiatric treatment are essential to prevent the recurrence of psychotic episodes and ensure long-term recovery. The control of substance abuse is crucial for the management of substance-induced psychosis in order to reduce the risk of severe self-harming behaviors.

## 5 Conclusion

This case highlights the severe implications of substance-induced psychosis, including extreme self-harm behaviors such as self-amputation of the auricles and penis. The successful management of this patient underscores the need for a multidisciplinary approach involving surgical, psychiatric, and psychological interventions. The patient’s strong motivation for penile reconstruction played a pivotal role in his adherence to treatment and abstinence from substance use, ultimately contributing to positive outcomes. Continuous follow-up and comprehensive care are essential to address the psychiatric and substance abuse dimensions of such complex cases, ensure long-term recovery, and prevent recurrence.

## Data Availability

The original contributions presented in the study are included in the article/supplementary material. Further inquiries can be directed to the corresponding author.
